# Enhancing metasurface fabricability through minimum feature size enforcement

**DOI:** 10.1515/nanoph-2024-0150

**Published:** 2024-05-21

**Authors:** Pavel Terekhov, Shengyuan Chang, Md Tarek Rahman, Sadman Shafi, Hyun-Ju Ahn, Linghan Zhao, Xingjie Ni

**Affiliations:** Department of Electrical Engineering, 183843 The Pennsylvania State University, University Park, PA 16802, USA

**Keywords:** metalens, metasurface, nanofabrication, fabrication-friendly

## Abstract

The metasurfaces have shown great potential for miniaturizing conventional optics while offering extended flexibility. Recently, there has been considerable interest in using algorithms to generate meta-atom shapes for these metasurfaces, as they offer vast design freedom and not biased by the human intuition. However, these complex designs significantly increase the difficulty of fabrication. To address this, we introduce a design process that rigorously enforces the fabricability of both the material-filled (fill) and empty (void) regions in a metasurface design. This process takes into account specific constraints regarding the minimum feature size for each region. Additionally, it corrects any violations of these constraints across the entire device, ensuring only minimal impact on performance. Our method provides a practical way to create metasurface designs that are easy to fabricate, even with complex shapes, hence improving the overall production yield of these advanced meta-optical components.

## 1 Introduction

Metasurfaces have attracted widespread interest across various application areas, owing to their versatility and the variety of degrees of freedom they provide for controlling light [1], [2], [3], [4]. They have enabled the creation of optical components using modern nanofabrication technologies. Typically, to achieve desired optical responses from a metasurface, designers have focused on altering the shapes of its building blocks – known as meta-atoms. These meta-atoms, which may take forms such as rectangles, disks, or concentric rings, are tailored along with the material properties to achieve the desired outcomes [5], [6], [7], [8], [9], [10], [11], [12], [13]. As interest in multifunctional metasurfaces grows, for example, in controlling amplitude, dispersion, and polarization, designers are incorporating increasingly complex shapes into their designs [14], [15], [16]. In addition, another important aspect of metasurface advancement for commercial markets is the challenge of large-scale fabrication and mass production [17], [18], [19], [20], [21], [22], [23], [24], [25].

Recently, a notable shift has occurred towards algorithm-generated, free-form meta-atom shapes. This method significantly broadens the design possibilities for metasurfaces and reduces biases that may arise from human intuition during the design process [26], [27], [28], [29], [30], [31]. However, this complexity presents greater challenges in the fabrication of these devices. It is crucial to consider the limitations of the fabrication technology to be used during the design process to ensure the metasurfaces can be manufactured [32], [33]. There are studies that incorporate fabrication feasibility consideration into the inverse design processes [34], [35]. In those studies, the electromagnetic structure design is integrated with fabrication feasibility during the optimization process. This integration can be managed at the meta-atom level, but it is challenging at the device level, often limiting the size of the device. Previously, we also introduced an algorithm that generates free-form meta-atoms while adhering to minimum feature sizes (MFS) [36]. However, this approach only enforces MFS at the individual meta-atom level. Compliance at this level does not guarantee that a metasurface, comprising various closely positioned meta-atoms, will also meet these requirements. Moreover, some algorithms for generating meta-atoms lack MFS enforcement at all.

Addressing this issue, we have developed a universal algorithm that checks and adjusts metasurface designs at the entire device level, based on any specified MFS requirements. Our approach implements a multi-level optimization process that separates the electromagnetic structure design process from the fabrication feasibility optimization. We retain the computationally intensive electromagnetic structure design process at the meta-atom level, while we enforce MFS requirements and correct the layout design at the device level. At this level, we consider only the effective properties of each meta-atom. This approach significantly reduces the computational power needed, enabling us to design and correct large-scale metasurface devices. This offers a holistic solution to the challenges mentioned above.

## 2 Results and discussion

**Figure 1:**
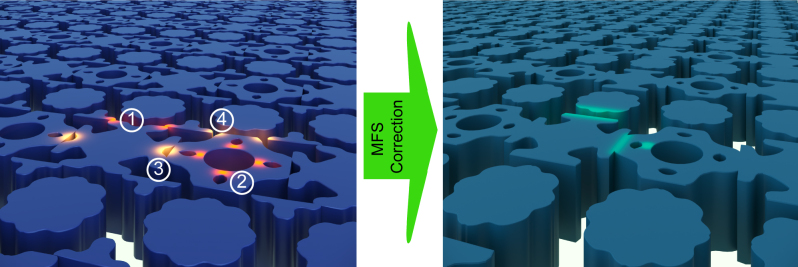
An artistic depiction illustrating various cases of MFS violations. Regions where the ‘fill’ requirements are not met are highlighted in red and labeled as ① and ②, while regions indicating ‘void’ requirement violations are marked in orange and labeled as ③ and ④. The regions that have been corrected in the design are distinctly marked in cyan for easy identification.

In our approach, we consider metasurface designs that consist of various meta-atoms, each with a uniform height and a binary material composition. Each meta-atom is defined by a set of polygons, labeled either ‘fill’ (material fill) or ‘void’ (empty space). To effectively check minimum feature size (MFS) requirements in such designs, our algorithm employs a ‘rolling circle’ method. This involves a circle, with a radius equal to the specified MFS, moving in a way that its center traces along the edges of each polygon. The algorithm tracks the number of times the circumference of this circle intersects with edges of adjacent polygons. These intersections help identify areas where the design does not meet MFS requirements. During the checking sequence, the algorithm separately assesses the ‘fill’ and ‘void’ areas along with their respective MFS requirements (MFS_f_ and MFS_v_). MFS_f_ defines the minimum fabricable size for material regions. Conversely, MFS_v_ defines the minimum fabricable size for voids. Figure 1 illustrates a metasurface layout with four possible cases of MFS violations and its corresponding corrected version.

**Figure 2:**
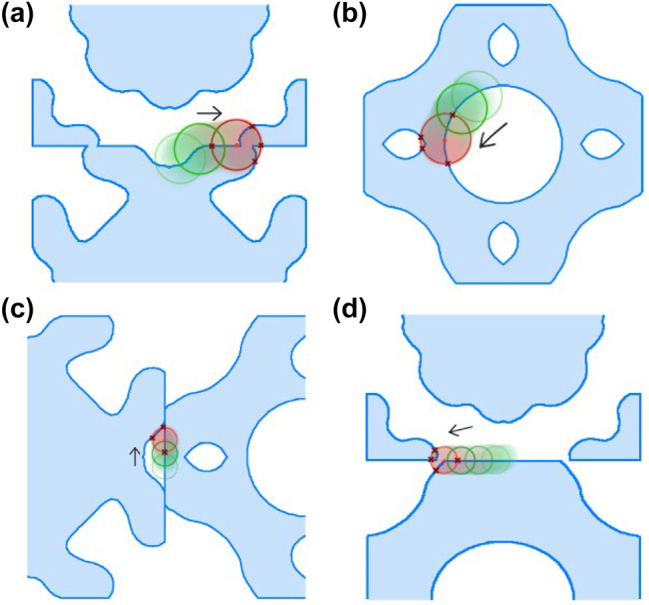
An illustration of the four steps in the ‘rolling circle’ method for assessing the fabricability of metasurface designs. (a) Step 1: A rolling circle with a radius *R* = MFS_f_ evaluates the fabricability of a single fill region. (b) Step 2: A rolling circle with a radius *R* = MFS_v_ evaluates the fabricability of a single void. (c) Step 3: A rolling circle with a radius *R* = MFS_v_ checks the distance between two fill regions. (d) Step 4: A rolling circle with a radius *R* = MFS_f_ checks the distance between two voids.

Our algorithm operates in four distinct steps, as illustrated in Figure 2. First, it checks the fabricability of each fill polygon independently. A ‘rolling circle’ with a radius *R* = MFS_f_ moving along the edges of the fill polygon. If the number of intersections between the circle and the polygon is not exactly two, the shape is considered non-fabricable. Specifically, four intersections indicate that the polygon at the current location is too small for fabrication, while zero intersections suggest that the entire polygon is smaller than MFS_f_, rendering it non-fabricable. Second, in a similar way, the algorithm checks the fabricability of each void polygon. This is done by traversing its edges with a ‘rolling circle’ having a radius *R* = MFS_v_. Third, the algorithm assesses the distances between different fill polygons. It uses a ‘rolling circle’ with a radius *R* = MFS_v_ to traverse each fill polygon. If there is at least one intersection with any other fill polygon, the shape is deemed non-fabricable. The algorithm marks previously checked fill polygons to exclude them from further consideration. Fourth, the algorithm checks the distances between any two void polygons within one fill polygon, as well as the distances between each void polygon and the edges of the fill polygon where it resides. This is done using the same approach with a ‘rolling circle’ of radius *R* = MFS_f_. All the possible cases with their corresponding numbers of intersections are listed in Table 1. Through these four steps, our algorithm thoroughly evaluates any metasurface layout containing multiple meta-atoms to ensure compliance with both fill and void MFS requirements.

**Table 1:** The assessment of design fabricability based on the number of intersections encountered during various ‘rolling circle’ evaluation steps. The green circles have a radius of MFS_f_ and the orange ones have a radius of MFS_v_.

Step	Illustration	# of intersections	Fabricable
1, 2	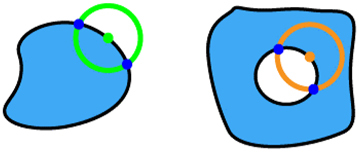	2	Yes
	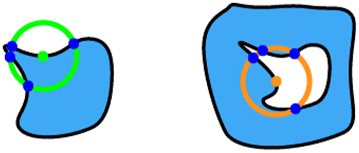	4	No
	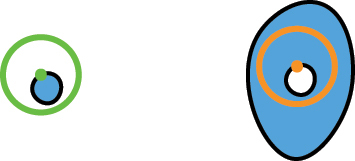	0	No
3, 4	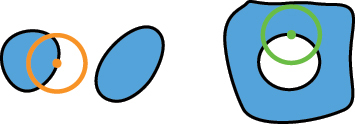	0	Yes
	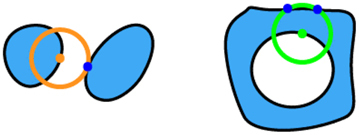	≥1	No

Our algorithm is not limited to checking individual meta-atom designs but is also capable of checking the entire layout of a metasurface design consisting of a large number of different meta-atoms. Furthermore, our algorithm is capable of replacing the meta-atoms that are found non-fabricable in the metasurface layout. To demonstrate this, we design a metasurface by placing meta-atoms from a predefined meta-atom library based on the desired optical responses (*e.g.*, phase, transmission, etc.) at each spatial position.

**Figure 3:**
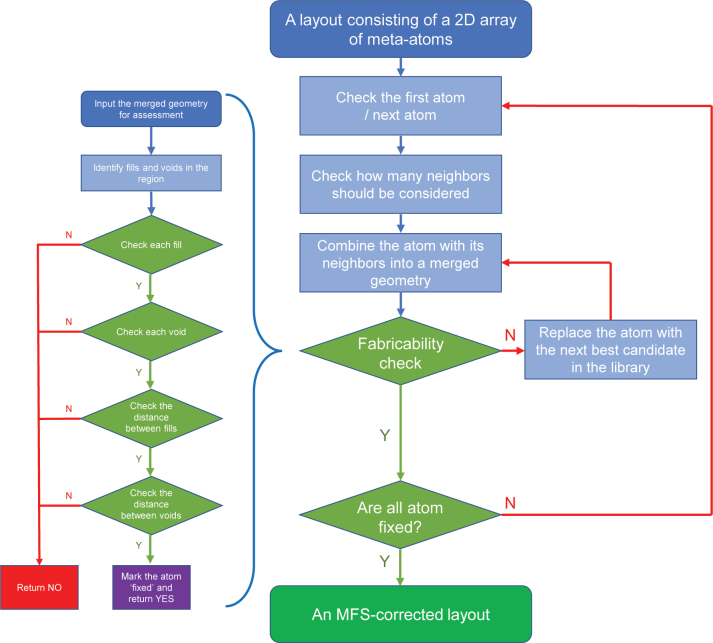
A detailed flowchart illustrates the workflow of the algorithm.

We first merge several meta-atoms and assess the fabricability of the resultant merged geometry. Beginning from one corner of the layout, the algorithm sequentially inspects the meta-atoms in a predefined order (in our case, from left to right and top to bottom). If a meta-atom meets the MFS requirement, the algorithm marks it as ‘fixed’ and excludes it from further consideration. In the event of a violation of the MFS requirement, the algorithm substitutes the meta-atom under assessment with the next best candidate from the meta-atom library. Figure 3 presents the algorithm’s workflow.

**Figure 4:**
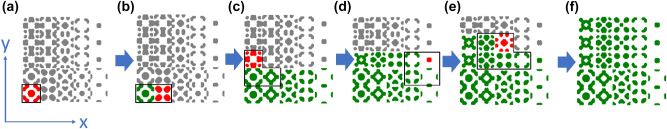
The process of checking layout fabricability. The check begins from the bottom left corner and proceeds along the *x*-axis; after completing a row, the algorithm moves to the leftmost atom in the row above. (a) A single meta-atom is checked for fabricability. (b–e) Four different scenarios for different meta-atom positions in the layout. (f) The final metasurface layout, where all meta-atoms are fixed. In (a–e), the meta-atoms under assessment are marked in red, fixed meta-atoms in green, and those yet to be checked in grey.

The process is illustrated in Figure 4, which depicts a metasurface layout consisting of free-form meta-atoms arranged in a square grid. The first meta-atom in the layout is evaluated for fabricability on its own, as shown in Figure 4(a). Following this, four scenarios (Figure 4(b)–(e)) may be considered in different areas of the layout. It is important to note that when assessing a meta-atom, all its adjacent fixed meta-atoms (including diagonal neighbors) must also be considered. In the bottom boundary of the layout, only two atoms need to be simultaneously checked each time (Figure 4(b)). For each meta-atom on the left boundary of the layout, three meta-atoms need to be checked each time, as the meta-atom under assessment is adjacent to two fixed meta-atoms (Figure 4(c)). For each meta-atom on the right boundary, four meta-atoms need to be checked simultaneously since it is adjacent to three fixed meta-atoms (Figure 4(d)). Lastly, for any meta-atoms in the middle of the layout, simultaneous consideration of five atoms is needed (Figure 4(e)). Our algorithm terminates once all the meta-atoms in the metasurface layout are fixed (Figure 4(f)). It also records the fabricability of each specific combination of meta-atoms that have been assessed, allowing for reuse to accelerate the process.

**Figure 5:**
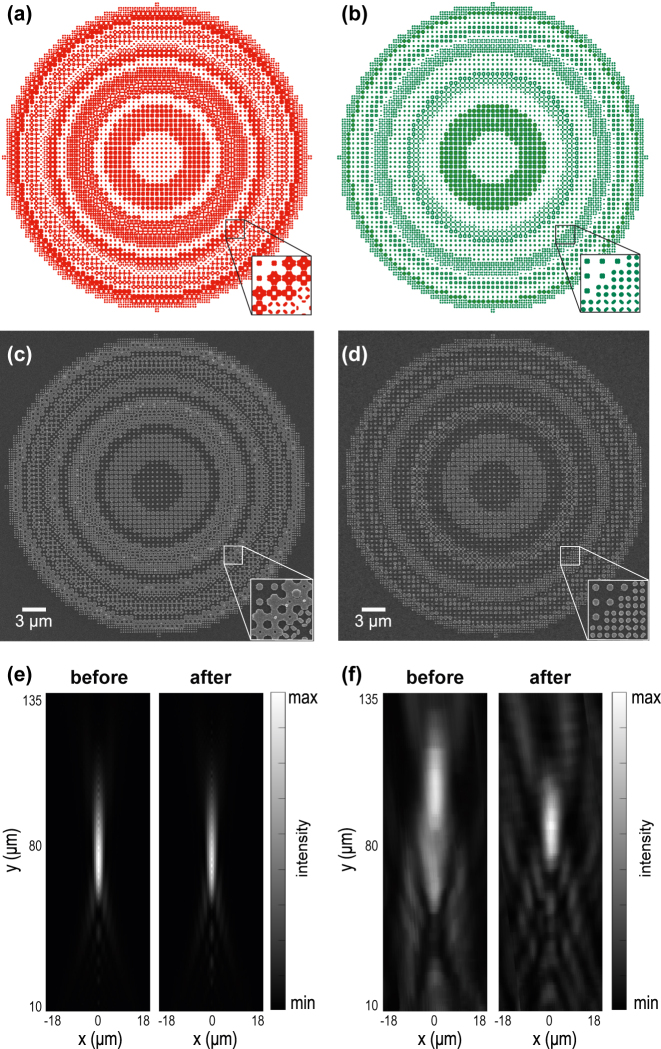
The layouts, fabricated metalenses, and performance evaluation before and after correction. (a, b) The layouts of the metalenses before and after correction. (c, d) SEM images of fabricated metalenses before and after correction. The insets in (c) and (d) showcase the same areas as the insets in panels (a) and (b). (e) Comparison of the focusing performance of the ‘ideal’ metalenses before and after correction numerically calculated using beam propagation method. The metalenses show similar performance with ‘after correction’ metalens being just slightly worse. (f) Comparison of experimentally measured performance of the fabricated metalenses before and after correction. The ‘before correction’ metalens shows the degraded performance.

As a demonstration, we designed a metalens composed of free-form silicon meta-atoms on a glass substrate. The metalens with a diameter of 40 μm is designed to focus light at 80 μm for a wavelength of 1064 nm. The layout underwent processing by our algorithm, considering practical fabrication constraints. The lattice constant of the layout is 600 nm, with the MFS requirement set at 100 nm for both fills and voids. We fabricated the silicon metalenses using the layouts before and after MFS correction (Figure 5(a) and (b)). The proximity effect correction was additionally used for both layouts’ fabrication to confirm that the non-fabricable layout cannot be corrected simply by existing means. The scanning electron microscope (SEM) images of the resulting metalenses (Figure 5(c) and (d)) reveal that non-fabricable regions led to patterns that stuck together, as small features were not successfully resolved during the fabrication process. In contrast, with MFS correction, the meta-atoms were successfully resolved, and fabrication quality increased dramatically. We highlight that the MFS correction had a minimal impact on the metalens’s performance degradation. Our numerical simulations (Figure 5(e)) confirm that the performance of the corrected metalens only slightly decreased. In contrast, the experimental results show significant differences between two metasurfaces. As presented in Figure 5(f), the light is more tightly focused by the corrected metalens; additionally, its focal distance is in good agreement with the prediction. In turn, the fabrication defects lead to the poor performance of not corrected metalens: its focal distance is significantly shifted, and the focal spot and depth of focus are broadened that means that the device does not exhibit the designed properties. These experimental results confirm that the proposed correction algorithm can ensure the optimal performance of fabricated metasurfaces.

**Figure 6:**
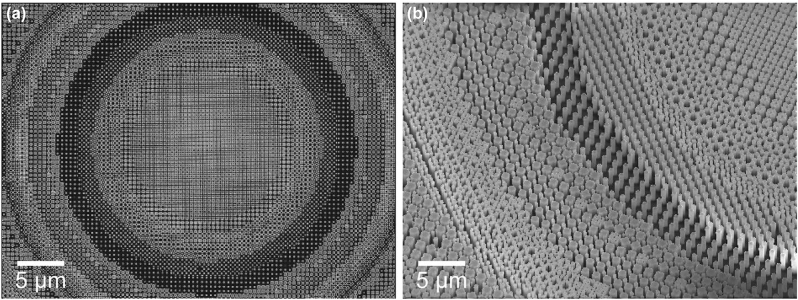
SEM images of an MFS-corrected metalens consisting of freeform meta-atoms. The minimum feature size requirements are strictly met, enabling maximal use of geometrical degrees of freedom. (a) Top view. (b) Tilted view.

As another example for our approach, Figure 6 showcases another metasurface fabricated using the layout MFS correction. It demonstrates that complex free-form shapes, including those with high-aspect-ratio features (up to 26:1), are all well-resolved. The fabrication of high aspect-ratio structures at the nanoscale remains challenging [37] but highly desirable for the wavefront manipulation since aheight increase provides more freedom for phase manipulation. Successfully manufacturing high aspect ratio freeform metalenses is promising for overcoming chromatic aberrations [38] and generally expanding the functionality of meta-devices [39].

## 3 Discussion

We introduced and demonstrated an approach for rigorous minimum feature size (MFS) enforcement to create fabrication-friendly metasurface designs. We outlined a four-stage process for inspecting and correcting arbitrary layouts of metasurface designs to verify and ensure their fabricability. To illustrate the improvements in fabricability, we fabricated and experimentally characterized metalenses with layouts both before and after applying the MFS correction using our algorithm. We showed that the ideal metalenses with and without MFS correction display comparable performance; however, the fabricated metalens without MFS correction experiences significant performance degradation due to its non-fabricable features.

We note that the proposed algorithm constitutes an additional step in the design phase, where the resulting corrected layouts can be further processed with proximity effect correction (PEC). PEC is widely used during fabrication phase to ensure that the manufactured patterns match the intended layouts. However, PEC does not incorporate information about device performance. In contrast, our algorithm offers a complementary approach to adjust the layout before PEC, replacing problematic regions with fabrication-friendly alternatives without compromising performance.

Our approach empowers the photonics community to fully leverage the potential of complex, non-intuition-biased meta-atom designs without compromising fabricability, which paves a way to meta-devices with improved efficiency and new functionalities. It can be particularly useful for mass-production fabrication methods such as photolithography and nanoimprinting, where the minimum feature size is more critical compared to what can be achieved by electron beam writing.
